# The Gothic Furnace

**Published:** 1874-12

**Authors:** 


The Gothic Furnace.—We looked into the merits of this excellent heating device last month, and expressed our opinion of it. Since that time we have had some additional evidence of its excellence. We heard that one of these furnaces was used in heating the great establishment of Baldwin, the Clothier, corner of Broadway and Canal Street. So we had a talk with Mr. Baldwin concerning the furnace, and found him very favorably impressed with it. He said it had given out all the heat required, and that it consumed only a moderate amount of coal. We looked at the furnace and found it in excellent condition, after its nine years' wear, and destined, in all probability, to last a lifetime. It is a small-sized Gothic, being No. 9, and costing

$25 when new. It heats the large store, 50 x 100 feet, all through the winter, and runs along, year after year, as regularly as clock-work. After this evidence, we do not wonder that Prof. Romer, of the N. Y. Free Academy, testifies that "the Gothic is the best furnace in the world." Its great advantage appears to be in the fact of its good-sized fire-pot and its immense gothic dome, which gives an unusually large radiating surface. The testimony of the porter at Baldwin's is to the effect that it is never necessary to heat the fire-pot to exceeding redness, as the very large radiating surface secures the heating of all the air which can be forced into the structure surrounding the furnace. We can find no furnace in this market which equals this; and as it is constructed to burn coal or wood, and of numerous sizes, both for dwelling and public edifices, we think the needs of all persons and localities can be fully met. We don't know that we can do better than to advise all our readers who are looking for an excellent heating-device, to correspond with Mr. A. M. Lesley, 224 and 226 West 23d Street, who is the manufacturer.

